# Development of a Stair-Step Multifrequency Synchronized Excitation Signal for Fast Bioimpedance Spectroscopy

**DOI:** 10.1155/2014/143461

**Published:** 2014-02-19

**Authors:** Yuxiang Yang, He Bian, Fangling Du, Qiang Sun, He Wen

**Affiliations:** ^1^Department of Precision and Instrumentation Engineering, Xi'an University of Technology, Xi'an 710048, China; ^2^Department of Electronic Engineering, Xi'an University of Technology, Xi'an 710048, China; ^3^Department of Instrumentation Science and Technology, Hunan University, Changsha 410082, China

## Abstract

Wideband excitation signal with finite prominent harmonic components is desirable for fast bioimpedance spectroscopy (BIS) measurements. This work introduces a simple method to synthesize and realize a type of periodical stair-step multifrequency synchronized (MFS) signal. The Fourier series analysis shows that the *p*-order MFS signal *f*(*p*, *t*) has constant 81.06% energy distributed equally on its *p*  2^*n*^th primary harmonics. The synthesis principle is described firstly and then two examples of the 4-order and 5-order MFS signals, *f*(4, *t*) and *f*(5, *t*), are synthesized. The method to implement the MFS waveform based on a field-programmable gate array (FPGA) and a digital to analog converter (DAC) is also presented. Both the number and the frequencies of the expected primary harmonics can be adjusted as needed. An impedance measurement experiment on a RC three-element equivalent model is performed, and results show acceptable precision, which validates the feasibility of the MFS excitation.

## 1. Introduction

Measurement of some kind of physical parameters over a broad frequency range, a technique called spectroscopy measurement, is very popular in modern scientific researches and engineering applications. For example, bioimpedance spectroscopy (BIS) technology, which performs measurement of complex electrical impedance of biomaterials over a certain frequency range, can provide valuable information about the physiological status of living tissues [1]. In recent years, BIS technology has been increasingly wide studied and adopted in clinical diagnoses on tissue ischemia [2, 3], cancer [4, 5], and radiation sequelae [6].

The most commonly used BIS measurement technique is the traditional frequency-sweep approach which performs measurement of impedance at a single frequency point in a time and sweeps the specific frequencies in the range of interest [7, 8]. The main characteristic of frequency-sweep is the relatively long measuring time [9, 10]. However, the physiological status of living body, such as the cardiovascular system, is dynamic because of its blood flow, heartbeat, and other factors. The frequency-sweep method actually measures the steady-state response of biological time-varying systems, and the dynamic behavior is usually removed from the impedance signal by means of averaging [11, 12]. So the BIS data based on frequency-sweep measurement technology cannot accurately represent the instantaneous impedance spectra and may lose important diagnostic information [13, 14].

In recent years, the multifrequency simultaneous measurement technique, which applies a broadband excitation and gains its resulting frequency response by means of spectral analysis using the Discrete Fourier Transform (DFT) algorithm, has been becoming popular for time-varying bioimpedance [10, 12, 15, 16]. Compared to the frequency-sweep approach, this fast BIS approach can reduce measuring time drastically by obtaining the impedance frequency response at different frequencies simultaneously and will be highly desirable for time-varying biological systems, such as the respiratory or cardiovascular systems [11, 17].

Excitation signal with wide spectrum is a crucial part for multifrequency simultaneous measurement of BIS. Maximum length sequences (MLS) [18] and chirp pulse [19] have been proposed as excitation signals for fast BIS measurements. The spectra of MLS and chirp pulse both have a continuous plateau envelope where most of the energy is concentrated. But in most BIS applications, only a discrete set of frequencies is required to fit the impedance data to a model like Cole equation [20], and BIS devices usually need to perform measurements at several to tens of isolated frequencies (usually with large frequency intervals) over a wide range of frequencies (often from 5 kHz to 1 MHz) [21]. Consequently, wideband excitation signal whose energy is distributed mainly in finite frequency points with large frequency spacing is ideal for BIS measurements.

The authors previously proposed a method based on Walsh functions to synthesize multifrequency mixed (MFM) signal [22, 23], which is a type of binary sequence with only +1 and −1 value and has majority of energy concentrated on its 2^*n*^th primary harmonics. But the MFM signal has several drawbacks. Firstly, the number of primary harmonics is limited to odd numbers to avoid the appearance of the third value “0” besides “+1” and “−1.” Secondly, the amplitudes of the primary harmonics in a MFM signal are not equal and decrease as harmonic order increases. Furthermore, the power percentage sum of the primary harmonics in a MFM signal is not very high and has a downward trend as the number of primary harmonics increases from 73.91% in the five-frequency mixed signal *f*(5, *t*) down to 68.90% in *f*(7, *t*) and down to 65.52% in *f*(9, *t*) [22].

This paper introduces an improved synthesis method based on Walsh functions to generate a type of stair-step multifrequency synchronized (MFS) signal. The MFS signal is still a periodical waveform but has an increased and constant 81.06% energy distributed equally on its 2^*n*^th primary harmonics. The restriction on the number of primary harmonics is also nonexistent in the MFS signals. The rest of the paper is organized as follows.  Section 2 describes the synthesis methods of the MFS signal based on Walsh functions, and Section 3 analyzes the spectral characteristic of the MFS signal based on the Fourier series theory.  Section 4 introduces briefly a circuit realization approach of the MFS signal, and Section 5 performs an impedance measurement experiment on a RC three-element equivalent model to test the feasibility of the MFS signal. Finally, the advantage and disadvantage of the MFS signal are discussed in Section 6.

## 2. Synthesis of the MFS Signal

Like the MFM signal, the MFS signals are also synthesized based on the Walsh functions, which already have detailed description in [22]. Similarly, we can synthesize a MFS signal based on simple superposition of the Walsh functions SAL(2^*k*−1^, *t*) in accordance with

(1) $$\begin{array}{c} f\left(p,t\right)=\sum_{k=1}^{p}\text{SAL}\left(2^{k-1},t\right), \end{array}$$

where *p* is the order of the MFS signal and also denotes the number of primary harmonics in *f*(*p*, *t*).

Let *p* = 4; then we can get the 4-order MFS signal:

(2) f(4,t)=SAL(20,t)+SAL(21,t)+SAL(22,t)+SAL(23,t).

**Figure 1:**
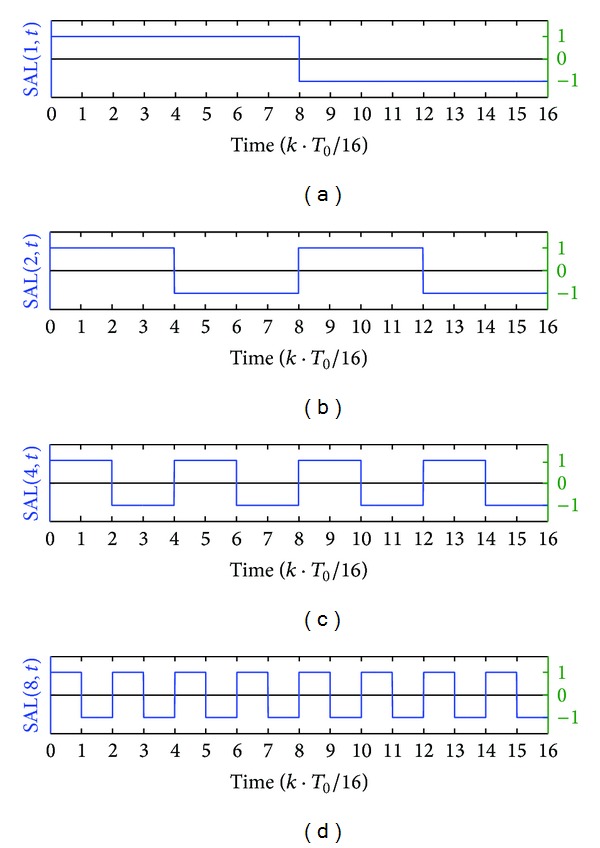
The Walsh functions SAL(2^0^, *t*) to SAL(2^3^, *t*) in a period.

The four Walsh functions SAL(2^0^, *t*) to SAL(2^3^, *t*) used to compose *f*(4, *t*) are shown in Figure 1, in which each Walsh function is equally divided into 16 components (code elements) in a period *T*_0_. So each function can be expressed by a row vector with 16 elements which have only two possible values +1 and −1.

The resultant vector *f*(4, *t*) can be expressed as

(3) $$\begin{array}{c} f\left(4,t\right)=\left[4,2,2,0,2,0,0,-2,2,0,0,-2,0,-2,-2,-4\right]. \end{array}$$

**Figure 2:**
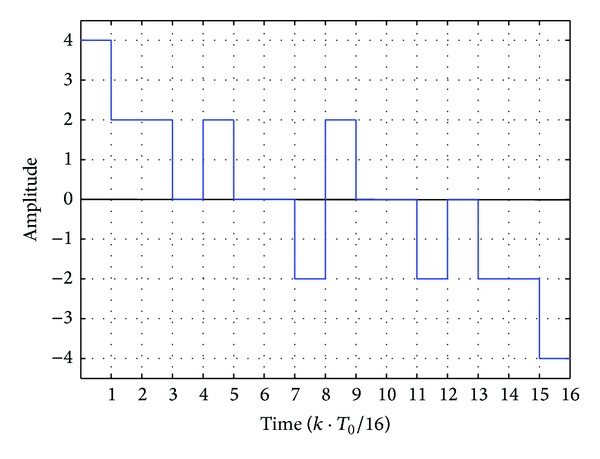
Waveform of the synthesized 4-order MFS signal *f*(4, *t*) with 16 code elements in a period.

The waveform of the synthesized 4-order MFS signal *f*(4, *t*) is shown in Figure 2.

## 3. Spectrum Analysis of the MFS Signal

Similar to the MFM signal as mentioned in [22], the MFS signal *f*(4, *t*) also conforms to the Dirichlet conditions and can be decomposed into infinite sinusoidal harmonics:

(4) $$\begin{array}{c} f\left(4,t\right)=a_{0}+\sum_{k=1}^{\infty }a_{k}\cos k\omega_{0}t+\sum_{k=1}^{\infty }b_{k}\sin k\omega_{0}t, \end{array}$$

where

(5) $$\begin{array}{c} a_{0}=\frac{1}{T_{0}}\int_{T_{0}}f\left(4,t\right)dt \end{array}$$

(6) $$\begin{array}{c} a_{k}=\frac{2}{T_{0}}\int_{T_{0}}f\left(4,t\right)\cos k\omega_{0}t\;dt \end{array}$$

(7) $$\begin{array}{c} b_{k}=\frac{2}{T_{0}}\int_{T_{0}}f\left(4,t\right)\sin k\omega_{0}t\;dt. \end{array}$$

From Figure 2, we can see that the waveform of *f*(4, *t*) is an odd function and inversely symmetrical with respect to its middle point 8 in a period; then (5) and (6) are equal to 0; namely, *a*_0_ = 0, *a*_*k*_ = 0, and *b*_*k*_ in (7) can be converted into

(8) $$\begin{array}{c} b_{k}=\frac{2}{T_{0}}\int_{T_{0}}f\left(4,t\right)\sin k\omega_{0}t\;dt=\frac{4}{T_{0}}\int_{0}^{T_{0}/2}f\left(4,t\right)\sin k\omega_{0}t\;dt. \end{array}$$

Generally, if a periodic piecewise waveform has *m* + 1 discontinuities within [0, *T*_0_/2) at (0, *t*_1_,…, *t*_*m*_) *T*_0_/2^*n*^ with the corresponding amplitudes (*A*_0_, *A*_1_,…, *A*_*m*_), then the nonzero Fourier series coefficients *b*_*k*_ in (7) can be calculated by subsection integrations:

(9) bk=4T0∫0T0/2g(p,t)sinkω0t dt=4T0[∫0t1A0sinkω0t dt+∫t1t2A1sinkω0t dt  +⋯+∫tmT0/2Amsinkω0t dt]=2kπ[A0+(A1−A0)cos⁡kω0t1  +⋯+(Am−Am−1)cos⁡kω0tm−Amcos⁡kπ].

In Figure 2, *f*(4, *t*) has 6 discontinuities within [0, *T*_0_/2) distributed at (0,1, 3,4, 5,7)*T*_0_/16 with the respective amplitudes (4, 2, 0, 2, 0, −2). Then, according to (9), *b*_*k*_ can be computed as

(10) bk=2kπ(4−2cos⁡kπ8−2cos⁡3kπ8+2cos⁡4kπ8   −2cos⁡5kπ8−2cos⁡7kπ8+2(−1)k).

Also, according to Parseval theorem [24], the average power of *f*(4, *t*) in a period is equal to the total sum of the average power of its harmonic components:

(11) $$\begin{array}{c} P=\frac{1}{T_{0}}\int_{T_{0}}{\left|f(4,t)\right|}^{2}dt=a_{0}^{2}+\frac{1}{2}\sum_{k=1}^{\infty }(a_{k}^{2}+b_{k}^{2})=\frac{1}{2}\sum_{k=1}^{\infty }b_{k}^{2}, \end{array}$$

and the average power of the *k*th harmonic is

(12) $$\begin{array}{c} P_{k}=\frac{1}{2}b_{k}^{2}. \end{array}$$

**Figure 3:**
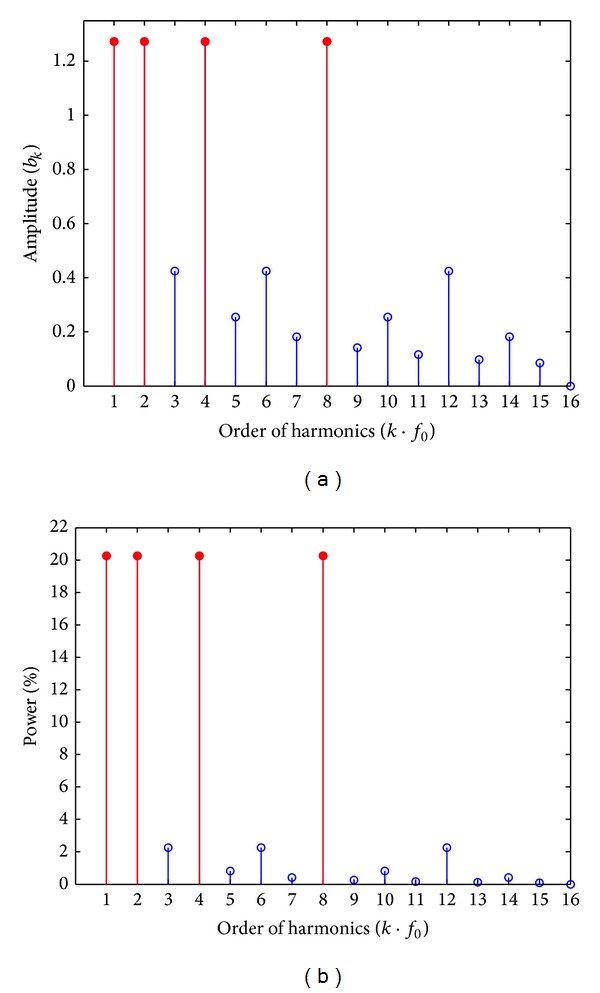
(a) Harmonic amplitude spectra *f*(4, *t*). (b) Harmonic power percentage of *f*(4, *t*).

According to the equation above, the first 16 harmonic amplitude spectra are calculated and shown in Figure 3(a), in which the expected 1st, 2nd, 4th and 8th primary harmonics, as marked with solid red color, have equal amplitude spectra. Also according to (11) and (12), the harmonic power percentage spectra of *f*(4, *t*) are calculated and shown in Figure 3(b), while the total power of *f*(4, *t*) is “4” which can be computed directly according to Figure 3 and (11).

Also, according to (4), *f*(4, *t*) can be represented by Fourier series:

(13) $$\begin{array}{c} f\left(4,t\right)=\sum_{k=1}^{\infty }b_{k}\sin k\omega_{0}t. \end{array}$$

The Fourier series expression in (13) indicates that the harmonic phase *φ*_*k*_ is either equal to 0 degree when *b*_*k*_ is positive or equal to 180 degrees when *b*_*k*_ is negative. So from Figure 3(a), we can estimate that the phases of the four 2^*n*^th primary harmonics are all equal to zero. The amplitudes, phases, and power percentage of the primary harmonics in *f*(4, *t*) are listed in Table 1, in which the power of the 4 expected primary harmonics is equal and their sum is up to 81.06% of the total power.

For another example, let *p* = 5; then the 5-order MFS signal *f*(5, *t*) can be expressed as

(14) f(5,t)=SAL(20,t)+SAL(21,t)+SAL(22,t) +SAL(23,t)+SAL(24,t)=[5,3,3,1,3,1,1,−1,3,1,1,−1,1,−1,−1,−3,3,1,1,  −1,1,−1,−1,−3,1,−1,−1,−3,−1,−3,−3,−5].

**Figure 4:**
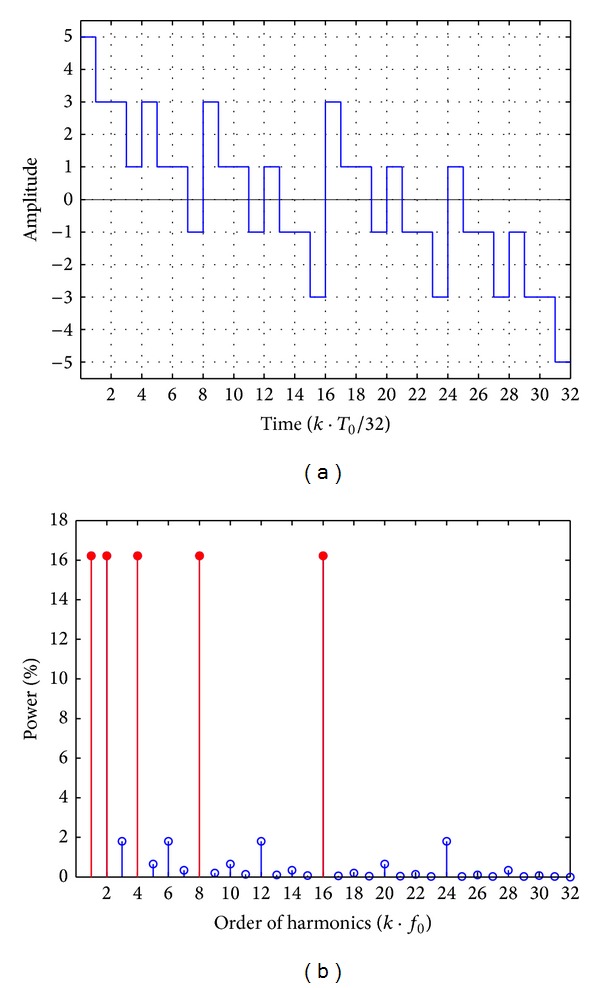
(a) Waveform of the 5-order MFS signal *f*(5, *t*) with 32 code elements in a period. (b) Harmonic power percentage of *f*(5, *t*).

The waveform of *f*(5, *t*) is shown in Figure 4(a), in which *f*(5, *t*) is composed of 32 code elements in a period *T*_0_ and is still an odd function and inversely symmetrical with respect to its middle point 16. Similarly, the amplitude spectra, phase, and power percentage of every harmonic in *f*(5, *t*) can be computed according to Fourier series theory mentioned above. The first 24 harmonic power percentages of *f*(5, *t*) are shown in Figure 4(b), and the five primary harmonics, namely, the 1st, 2nd, 4th, 8th, and 16th harmonic as marked with solid red color, have equal amplitude spectra. The amplitude, phase, and power percentage of the five primary harmonics are listed in Table 2, in which the power sum of the 5 primary harmonics is still equal to 81.06% of the total power.

There is an interesting rule that, for *p*-order MFS signal *f*(*p*, *t*), its *p* primary harmonics always have equal amplitude spectra and their power sum remains 81.06% of the total power. For another example, the 6-order MFS signal *f*(6, *t*) has six primary harmonics, namely, the 1st, 2nd, 4th, 8th, 16th, and 32th harmonic. Their amplitude, phase, and power percentages of the six primary harmonics are listed in Table 3, in which the power sum of the 6 primary harmonics is still equal to 81.06%.

From Tables 1, 2, and 3, we can conclude that the MFS signal *f*(*p*, *t*) has *p*2^*n*^th primary harmonic components with equal and prominent amplitudes which always occupy up to 81.06% of the total power. This characteristic will be beneficial to obtain higher signal-to-noise ratio (SNR) in BIS measurements with the MFS excitation. The primary harmonics could cover a large frequency range from *f*_0_ to 2^*p*−1^ *f*_0_ and the number of the expected primary harmonics *p* can be adjusted as needed in (1) in the synthesis procedure.

## 4. Realization of the MFS Excitation Source

**Figure 5:**
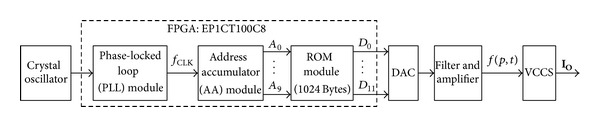
Block diagram of the hardware system to realize the MFS signal.

An excitation source based on the stair-step MFS signal is designed and realized, whose block diagram is shown in Figure 5. The major building block includes a field-programmable gate array (FPGA) platform, a digital to analog converter (DAC), and a voltage-controlled current source (VCCS).

The FPGA (Altera Corporation, San Jose, CA) is designed as function storage with parallel output for generation of the MFS signal, and the 12-bit DAC (AD9742, Analog Devices Inc., Norwood, MA, USA) converts the parallel data from FPGA into analog waveform. In the FPGA, a read-only memory (ROM) module is configured as a 12-bit memory with 1024 storage cells (words), in which a complete cycle of waveform data is stored sequentially. The 10 address lines of the ROM module, *A*_0_ ~ *A*_9_, are controlled by an address accumulator (AA) module which essentially is a 10-bit binary counter. When running, the AA module will count from 000H to 3FFH repeatedly under the counting clock from a phase-locked loop (PLL), and then the ROM module will output the corresponding digital waveform data addressing from 000H to 3FFH repeatedly at its 12-bit parallel data bus *D*_0_ ~ *D*_11_. Afterwards, the 12-bit DAC converts these 12-bit data into analog waveform, and ultimately waveform can be obtained after filtering and amplifying.

**Figure 6:**
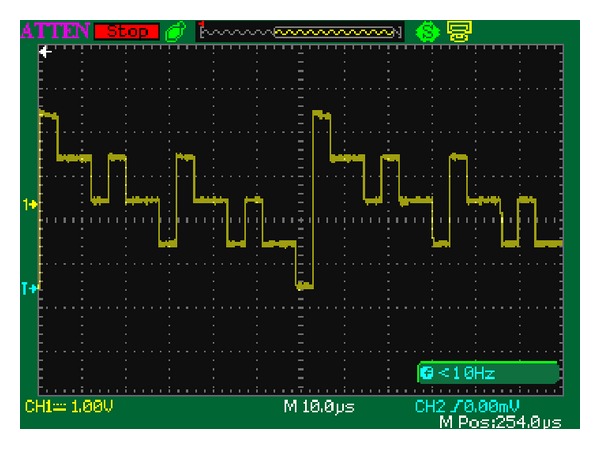
Oscillograph of *f*(4, *t*) (one more period).

A waveform example of *f*(4, *t*) is shown in Figure 6. The fundamental frequency of *f*(4, *t*) is 16 kHz since the clock frequency *f*_CLK_ from the PLL module is 16.384 MHz and the AA module completes a cycle addressing around the ROM module every 1024 clock. So the fundamental frequency of the output waveform can be computed as

(15) $$\begin{array}{c} f_{0}=\frac{1}{T_{0}}=\frac{1}{1024\times T_{\text{CLK}}}=\frac{f_{\text{CLK}}}{1024}=\frac{16384000}{1024}=16\;\left(\text{kHz}\right). \end{array}$$

The frequencies of the four primary harmonics in *f*(4, *t*), namely, the 1st, 2nd, 4th, and 8th harmonics, are 16 kHz, 32 kHz, 64 kHz, and 128 kHz. The fundamental frequency, as well as the other primary harmonics in MFS signal, can be adjusted as needed by changing the clock frequency *f*_CLK_ in the PLL module.

The VCCS is designed based on the current feedback amplifier (CFA) AD844 (Analog Devices, Norwood, MA) according to the method proposed by Bragos et al. [25], which has high output impedance and been widely adopted by many bioimpedance systems. The VCCS generates an output current **I**_**O**_ proportional to its input voltage, the MFS signal *f*(*p*, *t*).

## 5. Measurement Experiment

**Figure 7:**
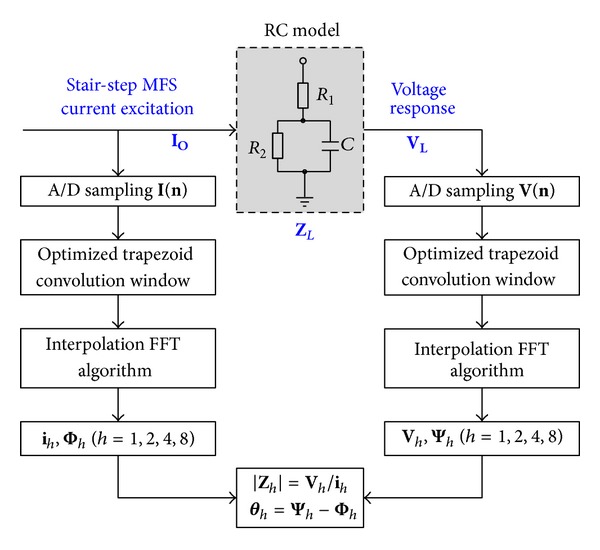
Impedance measurement scheme.

An impedance measurement experiment on a RC three-element equivalent model is designed to test the feasibility of the proposed MFS signal. The measurement scheme is shown in Figure 7, in which the excitation current source **I**_**O**_ is the MFS signal *f*(4, *t*). **I**_**O**_ flows through the load *Z*_*L*_, a RC three-element equivalent circuit model (dashed box in Figure 7), and gets the voltage drop **V**_**L**_. The practical RC model is manufactured, in which *R*_1_ and *R*_2_ are 1/4 watt metal-film resistors with ±1% tolerance, and *C* is a low-loss metalized polyester film capacitor with ±5% tolerance. In this experiment, we use the typical parameters *R*_1_ = 200 *Ω*, *R*_2_ = 200 *Ω*, *C* = 22 nF_°_.

**Figure 8:**
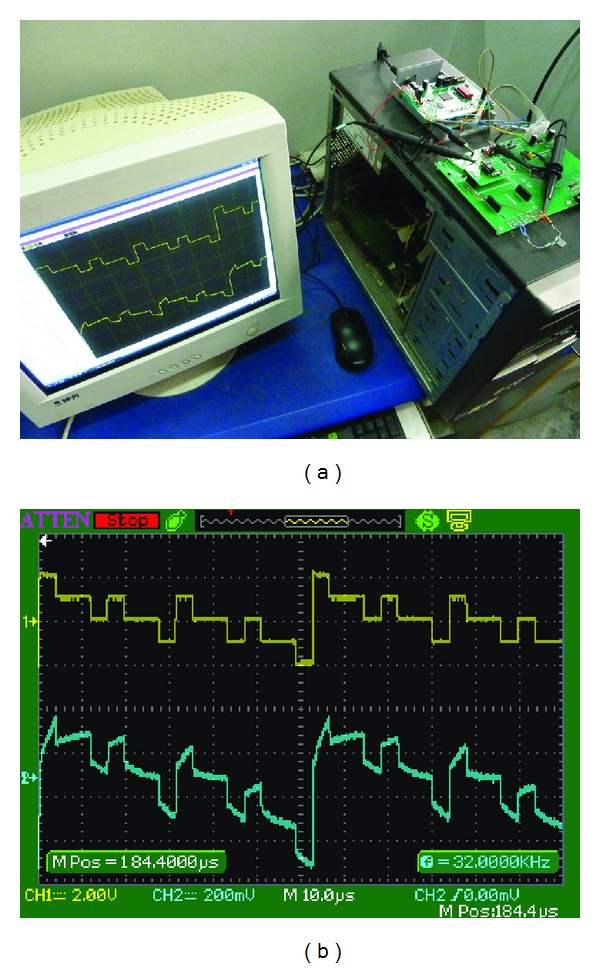
(a) Scene photo of measurement experiment. (b) Oscillograph of *f*(4, *t*) and its response voltage on a RC load.

The simultaneous sampling on **I**_**O**_ and **V**_**L**_ was performed by a data acquisition (DAQ) board PCI-50612 (Sichuan Top Measurement and Control Technology Co., Ltd., Chengdu, China), which is a PCI-bus based, 12-bit, four-channel synchronized DAQ board with as high as 50 MSps sampling rate. The DAQ board is inserted in the PCI slot of a PC computer, where the proprietary software TOPVIEW2000 runs as a human-machine interface, as shown in Figure 8(a).  Figure 8(b) displays the waveforms of the excitation current **I**_**O**_ and the response voltage **V**_**L**_ in an oscilloscope (Atten Electronics Co., Ltd., Shenzhen, China), where the upper waveform denotes **I**_**O**_, and the lower one denotes **V**_**L**_.

**I**_**O**_ and **V**_**L**_ were sampled simultaneously in the DAQ board and the corresponding discrete sequences **I**(**n**) and **V**(**n**) are obtained, respectively. **I**(**n**) and **V**(**n**) then were truncated using Optimized Trapezoid Convolution Window (OTCW) [26] to suppress spectral leakage and picket fence effects, and the corresponding interpolation FFT algorithm was performed on the two sequences, respectively. The primary harmonic amplitude and phase parameters of **I**(**n**) and **V**(**n**), namely, (**i**_*h*_, Φ_*h*_) and (**V**_*h*_, Ψ_*h*_) (*h* = 1,2, 4, 8), were obtained after FFT. Finally, the tested impedance amplitudes and phases are calculated according to the formula as follows:

(16) $$\begin{array}{c} Z_{h}=\frac{V_{h}}{i_{h}},\;\;\theta_{h}=\Psi_{h}-\Phi_{h}. \end{array}$$

The impedance measurement results at the four primary harmonic are shown in Table 4, in which **Z**_st_ denotes the standard impedance amplitudes, while ***θ***_st_ denotes the standard impedance phases, both measured by WK 6510B Precision Impedance Analyzer (Wayne Kerr Electronics Ltd., London, UK). **Z**_*h*_ and ***θ***_*h*_ are obtained impedance amplitudes and phases calculated according to (16). In Table 4, the mean relative error of the impedance amplitudes is 0.23%, while the mean phase absolute error is 0.20°.

## 6. Discussion

In recent years, there emerge different multifrequency simultaneous excitation signals for fast BIS or time-varying bioimpedance, such as maximum length sequences (MLS) [18] and chirp [19]. Both MLS and chirp signals are both spectral dense signals, whose energy is distributed equally over the full measurement bandwidth in which the magnitude of each individual frequency component is low and therefore the initial SNR of the measurements is discounted. However, in practice there seldom occurs a need to measure at all frequencies within the measurement bandwidth. More often only measuring several to tens of distinct frequencies (usually with large frequency intervals) is satisfactory. Therefore, the spectral sparse signals whose energy concentrates on the expected limited spectral lines, such as the sparse spectrum sequence (SSS) [27], the authors' MFM signals [22], and the MFS signals proposed in this paper, are more reasonable. According to the literature [27], a sample of the SSS signal has 76.2% of the total signal energy distributed equally on its four harmonics (1, 3, 5, 7). For *p*-order MFS signal *f*(*p*, *t*), it has constant 81.06% energy distributed equally on its *p*2^*n*^th primary harmonics (1,2, 4,…, 2^*p*−1^). For example, the 9-order MFS signal *f*(*p*, *t*) has 9 primary harmonics (1,2, 4,8, 16,32,64,128,256), each of which has 9% energy.

Besides the frequency spacing, crest factor (CF), the ratio of peak values to the average value, is another measure of an excitation signal. Under the same peak value, excitation with lower CF means more energy located on spectral lines in the excitation spectrum. The binary signals, such as MLS, MFM, and SSS, have the minimal CF (equal to 1). For *p*-order MFS signal *f*(*p*, *t*), its CF can be computed:

(17) $$\begin{array}{c} \text{CF}=\frac{\max_{n\in [0,T_{0}]}\left|f\left(p,t\right)\right|}{\sqrt{1/T_{0}\int_{0}^{T_{0}}{\left|f(p,t)\right|}^{2}dt}}=\frac{p}{\sqrt{p}}=\sqrt{p}. \end{array}$$

According to (17), CF = 2 for the 4-order MFS signal *f*(4, *t*), while CF = 3 for the 9-order MFS signal *f*(9, *t*). Compared with the binary signals, the relatively higher CF may become the main drawback for the MFS signals. But in the situation when CF is not the main problem for a system, the MFS signal like *f*(9, *t*) could still be an attractive excitation signal because it covers a large frequency range.

Another severe drawback for all rectangular signals, including MLS, binary chirp, SSS, MFM, and MFS signals, is that the energy in rectangular signals is not solely located at the specific frequencies but inevitably spreads over a great number of undesired frequencies. The undesired frequency components, whose frequencies are higher than half of the data sampling frequency, will lead to spectrum aliasing when performing FFT operations [28], and will contribute to the ultimate measurement errors. So generally, an antialiasing filter is needed before sampling for the BIS measurement system with rectangular excitations.

Another reason that degrades the measurement precision is non-integer-period sampling for FFT operation, like the measurement scheme as shown in Figure 7. The DFT theory always assumes that the input sequence is periodic (assumed period extension), so if the input sequence finishes on a whole number of periods, everything is fine. Otherwise, discontinuity occurs in the assumed extended sequences and brings spectral leakage in FFT operation [29, 30]. Unfortunately, integer-period sampling is usually hard to achieve, since sampling device is hard to begin and end exactly at the signal's head and tail, respectively. The undesired effects of the spectral leakage can be suppressed by weighting the input sequence by a suitable window [31], as we do in this paper, at the cost of increased computation load. Recently, Sanchez et al. [32] show us a successful system where the excitation signal generation and data sampling are under the control of the same clock in FPGA, which achieves integer-period sampling and avoids spectral leakage. So, we have reasons to believe that such integer-period sampling based BIS system will work much better for the proposed MFS excitation and also for other rectangular excitation signals.

## 7. Conclusion

Wideband excitation signal with sparsely distributed spectrum is desirable for fast BIS measurements. This paper introduces a simple method to synthesize and realize a type of MFS signal, which is a periodical stair-step waveform and has a constant 81.06% energy sparsely distributed equally on its 2^*n*^th primary harmonics. Compared with the previous MFM signal which has a downtrend on harmonic amplitudes, the MFS signal may be more favorable to get higher SNR at the expected 2^*n*^th primary harmonics because they have equal and prominent amplitudes. The primary harmonics could cover a large frequency range from the fundamental frequency *f*_0_ to 2^*p*−1^ *f*_0_ and the number of the expected primary harmonics *p* can be adjusted as needed. Furthermore, the MFS signal can be realized easily by FPGA + DAC, and the fundamental frequency *f*_0_, as well as the frequencies of other primary harmonics, can also be adjusted as needed in FPGA. Based on the features mentioned above, the proposed MFS signal may be a good choice of multifrequency excitation signal for fast measurement of BIS, and it also has a commendable reference value for other spectroscopy technologies such as electrochemical impedance spectroscopy (EIS).

## Figures and Tables

**Table 1:** Spectrum characteristics of the primary harmonics in the 4-order MFS signal *f*(4, *t*).

*H*_*k*_	*f*_0_	2*f*_0_	4*f*_0_	8*f*_0_	Sum
*b*_*k*_	1.27	1.27	1.27	1.27	
*P*_*k*_ (%)	20.26	20.26	20.26	20.26	81.06%
*φ*_*k*_	0	0	0	0	

**Table 2:** Spectrum characteristics of the primary harmonics in the 5-order MFS signal *f*(5, *t*).

*H*_*k*_	*f*_0_	2*f*_0_	4*f*_0_	8*f*_0_	16*f*_0_	Sum
*b*_*k*_	1.27	1.27	1.27	1.27	1.27	
*P*_*k*_ (%)	16.21	16.21	16.21	16.21	16.21	81.06%
*φ*_*k*_	0	0	0	0	0	

**Table 3:** Spectrum characteristics of the primary harmonics in the 6-order MFS signal *f*(6, *t*).

*H*_*k*_	*f*_0_	2*f*_0_	4*f*_0_	8*f*_0_	16*f*_0_	32*f*_0_	Sum
*b*_*k*_	1.27	1.27	1.27	1.27	1.27	1.27	
*P*_*k*_ (%)	13.51	13.51	13.51	13.51	13.51	13.51	81.06%
*φ*_*k*_	0	0	0	0	0	0	

**Table 4:** Results of impedance measurement experiment under the excitation of the MFS signal *f*(4, *t*).

Harmonics **H**_**k**_	Frequency (kHz)	Standard **Z**_st_	Measured **Z**_**h**_	Relative errors |**E**_**Z**_| (%)	Standard ***θ***_st_	Measured ***θ***_**h**_	Absolute errors |**E**_**P**_| (°)
*f*_0_	16	374.39	374.32	0.02	−11.48	−10.91	0.57
2*f*_0_	32	326.91	326.23	0.21	−17.53	−17.54	0.01
4*f*_0_	64	263.19	261.58	0.61	−18.73	−18.58	0.15
8*f*_0_	128	222.37	222.16	0.09	−13.50	−13.56	0.06

Mean error				0.23			0.20
